# Special Issue “Properties and Applications of Nanoparticles and Nanomaterials: 3rd Edition”

**DOI:** 10.3390/ijms27073023

**Published:** 2026-03-26

**Authors:** Xiaogang Li

**Affiliations:** Institute of Nuclear and New Energy Technology, Key Laboratory of Advanced Reactor Engineering and Safety of Ministry of Education, Tsinghua University, Beijing 100084, China; xiaogangli@mail.tsinghua.edu.cn

In recent years, we have witnessed the rapid evolution of nanomaterials, accompanied by a significant surge in scholarly and industrial interest. The emergence of diverse architectures—including nanoparticles, nano-grained alloys, and gradient nanostructures—facilitates the deployment of materials with exceptional properties in specialized practical environments. These materials find extensive applications across biochemistry or molecular medicine, fuel cells or metal–ion batteries, and flexible electronics, as well in various components related to energy. The performance of nanostructures is fundamentally dictated by their chemical composition and structural configuration, while also being sensitive to the synthesis and formation processes—factors critical to ensuring reliability in real-world applications.

The objective of this Special Issue is to establish a dedicated forum for scholarly discourse on the structure, properties, processing, and applications of nanoparticles and nanomaterials, with the aim of exploring novel avenues for addressing complex scientific challenges. The scope of this issue encompasses the aforementioned research domains, while also inviting contributions on adjacent topics, including the design of novel nanostructures and the functional modification of nanoparticles. Featuring contributions from distinguished academics and industrial researchers, this Special Issue presents cutting-edge and in-depth scientific insights intended to advance the collective understanding of critical issues in nanomaterials and to facilitate the resolution of these formidable challenges.

Carp et al. contributed with a paper entitled “Carbon Dots Meet MRI: Metal Doping for a Smart Contrast Agent Design”, which critically evaluates the potential of metal-doped carbon nanodots (C-dots) as versatile, potentially safer alternatives to conventional gadolinium-based MRI agents, analyzing how structural architecture—including dopant species, matrix positioning, and surface hydration—modulates *r*_1_ and *r*_2_ relaxivities. The featured research highlights the multifunctional potential of engineered C-dots while emphasizing that clinical translation remains contingent upon standardized relaxometry, scalable synthesis, and rigorous in vivo validation.

Daffalla contributed with a paper entitled “Biomass-Derived Magnetic Fe_3_O_4_/Biochar Nanoparticles from Baobab Seeds for Sustainable Wastewater Dye Remediation”, which reports the synthesis of baobab seed-derived, biochar-supported Fe_3_O_4_ nanoparticles (Fe_3_O_4_/BSB) as a high-performance, magnetically recoverable catalyst for the oxidative degradation of Congo red. The composite exhibits enhanced thermal stability and a porous architecture that facilitates 94.2% removal efficiency at pH 4, with kinetic data adhering strictly to a pseudo-second-order model. Furthermore, Fe_3_O_4_/BSB maintains significant catalytic activity over multiple cycles, presenting a sustainable and scalable solution for the remediation of dye-contaminated wastewater.

Kokina et al. contributed with a paper entitled “Using *Medicago sativa* L. Callus Cell Extract for the Synthesis of Gold and Silver Nanoparticles”, which demonstrates the rapid, eco-friendly biosynthesis of quasi-spherical gold and silver nanoparticles using *Medicago sativa* L. callus cultures as a sustainable reducing agent. Characterization via TEM and Nanoparticle Tracking Analysis confirmed the formation of polydisperse nanoparticles (5–25 nm core size), while elemental analysis verified successful metal reduction without exogenous contamination. These biogenic nanostructures offer a low-toxicity, cost-effective platform with significant potential for antibacterial and anticancer applications.

Aleksanyan et al. contributed a paper entitled “Effect of AgNPs on PLA-Based Biocomposites with Polysaccharides: Biodegradability, Antibacterial Activity and Features”, and it develops biodegradable PLA-based biocomposites with antibacterial silver nanoparticles and natural polysaccharides via twin-screw extrusion. Incorporating polysaccharides reduced elastic modulus and tensile strength but maintained elongation, while promoting soil biodegradation even with AgNPs reaching 29% mass loss. Despite AgNPs not inhibiting fungal growth, the materials retained bacterial inhibition and showed microbial biofouling, with chitosan enhancing degradation.

Zapałowska et al. contributed with a paper entitled “Impact of Silver Nanoparticles on the Gut Microbiota of the Earthworm Eisenia fetida”, which reveals that AgNP exposure induces substrate-dependent shifts in the gut bacterial communities of Eisenia fetida, with certain taxa declining while others persist, and alters trace element accumulation in earthworm tissues. These findings highlight the importance of considering both microbial and elemental responses when evaluating the environmental safety of nanotechnology in agricultural systems.

Mikheev et al. contributed with a paper entitled “Hybrid Fe_3_O_4_-Gd_2_O_3_ Nanoparticles Prepared by High-Energy Ball Milling for Dual-Contrast Agent Applications”. This study demonstrates the scalable synthesis of hybrid xGd_2_O_3_–(100 − x)Fe_3_O_4_ nanoparticles via high-energy ball milling for dual-contrast MRI applications. The milling process induces a phase transformation in Gd_2_O_3_ and reduces the magnetization of Fe_3_O_4_ due to surface spin effects. The resulting nanostructures exhibit high *r*_2_ relaxivity (up to 160 mM^−1^ s^−1^) and low *r*_1_ values, attributed to the specific crystallite sizes of both components.

This Special Issue showcases these emerging approaches and perspectives, with a commitment to advancing the effective application of nanoparticles and nanomaterials. We sincerely thank the authors who submitted manuscripts to our Special Issue, and we extend our sincere gratitude to our readers. We hope that this Special Issue will serve as a dynamic platform for discussion among researchers in the field of nanoparticles and nanomaterials. This Special Issue, “Properties and Applications of Nanoparticles and Nanomaterials”, has been held for three editions and has amassed a large number of high-quality papers. In light of the sustained submission of high-quality manuscripts on this topic, we are pleased to continue featuring this topic. Topics of interest include, but are not limited to, the structure, properties, processing, and applications of nanoparticles and nanomaterials, the design of novel nanostructures, and nanoparticle modification. Both original research articles and comprehensive reviews in these areas are warmly welcome.

